# The fifth vital sign? Nurse worry predicts inpatient deterioration within 24 hours

**DOI:** 10.1093/jamiaopen/ooz033

**Published:** 2019-08-28

**Authors:** Santiago Romero-Brufau, Kim Gaines, Clara T Nicolas, Matthew G Johnson, Joel Hickman, Jeanne M Huddleston

**Affiliations:** 1 Kern Center for the Science of Healthcare Delivery, Mayo Clinic, Rochester, Minnesota, USA; 2 Department of Nursing, Mayo Clinic, Rochester, Minnesota, USA; 3 Department of Surgery, Mayo Clinic, Rochester, Minnesota, USA; 4 Division of Hospital Internal Medicine, Department of Medicine, Mayo Clinic, Rochester, Minnesota, USA

**Keywords:** informatics, clinical deterioration, physiological pattern recognition, nursing, inpatient

## Abstract

**Introduction:**

Identification of hospitalized patients with suddenly unfavorable clinical course remains challenging. Models using objective data elements from the electronic health record may miss important sources of information available to nurses.

**Methods:**

We recorded nurses’ perception of patient potential for deterioration in 2 medical and 2 surgical adult hospital units using a 5-point score at the start of the shift (the Worry Factor [WF]), and any time a change or an increase was noted by the nurse. Cases were evaluated by three reviewers. Intensive care unit (ICU) transfers were also tracked.

**Results:**

31 159 patient-shifts were recorded for 3185 unique patients during 3551 hospitalizations, with 169 total outcome events. Out of 492 potential deterioration events identified, 380 (77%) were confirmed by reviewers as true deterioration events. Likelihood ratios for ICU transfer were 17.8 (15.2–20.9) in the 24 hours following a WF > 2, and 40.4 (27.1–60.1) following a WF > 3. Accuracy rates were significantly higher in nurses with over a year of experience (68% vs 79%, *P* = 0.04). The area under the receiver operator characteristic curve (AUROC) was 0.92 for the prediction of ICU transfer within 24 hours.

**Discussion:**

This is a higher accuracy than most published early warning scores.

**Conclusion:**

Nurses’ pattern recognition and sense of worry can provide important information for the detection of acute physiological deterioration and should be included in the electronic medical record.

## INTRODUCTION

Detecting when a patient is deteriorating is critical to hospital care. For example, two of the most common causes of acute inpatient deterioration are sepsis and acute respiratory failure, which have an in-hospital mortality of 20–30% and are involved in 34–52% of in-hospital deaths.^1^ These conditions show increased mortality if interventions are delayed.^2–4^

To improve the detection of acute inpatient deterioration, one solution is to use automated scores based on data from the electronic health record (EHR). Several scores using a combination of vital signs and other inputs, called early warning scores (EWS), have been developed to improve the recognition of inpatient physiological deterioration. However, EWS do not do not demonstrate accurate predictive capabilities when applied strictly,^5^ and to date they have failed to provide strong evidence of their ability to improve outcomes.^6^

EWS are being incorporated into the electronic medical record,^6–8^ and are used to inform the decision to activate rapid response teams (RRTs; a team of clinicians with specific expertise in responding to acutely deteriorating patients).^9–11^ Some of the most sophisticated EWS incorporate certain nursing assessments when using a data science approach, but they are generally limited to more objective assessments currently available in the EHR, such as neurological, skin, or nutritional status.^12^^,^^13^ While these approaches benefit from including a wider range of information not limited to vital signs or laboratory results, they are still missing an important piece of information that is currently not captured in the EHR: nursing assessment of patient risk of deterioration.

Despite this information not being routinely captured in the EHR, the most common criterion used to activate the RRT is the “worried criterion,”^14^^,^^15^ which is based on nurses’ pattern recognition. However, the predictive accuracy of nurses’ judgement of risk, whether based on analytical or intuitive pattern-recognition processes,^16^ has not been evaluated.

Our study aimed to evaluate whether the accuracy of nursing judgement, based on the “worried criterion” in detecting impending physiological deterioration merits its inclusion in the electronic medical record.

## METHODS

### Score development

**Figure 1.**
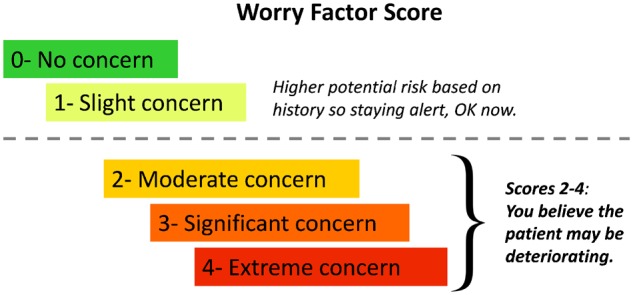
Worry Factor Score criteria. The Worry Factor Score is a very simple, 5-level scale. Scores 0 or 1 (above the dotted line) indicate the scoring nurse does not believe the patient is actively deteriorating. Scores of 2–4 (below the dotted line) indicate increasing level of worry or certainty that a patient is deteriorating.

As a way to collect nurses’ judgement, we first developed a score that would capture it. Ten focus group sessions involving frontline nursing staff were held in total. During the focus groups we developed the Worry Factor (WF) score, performed some preliminary testing using patient scenarios, and obtained buy-in from participating nurses. Approximately 150 staff members from two medical units and two surgical units participated. Despite initial consideration of a 7-point Likert-type scale, the final 5-point score described in Figure 1 was adopted based on the feedback received by frontline staff. Staff found it clearer to distinguish three levels for deteriorating patients (WF = 2, 3, or 4) and two for patients not in active deterioration.

### Collection of worry factor data

Nurses from two medical units (consisting of 50 total beds), and two surgical units (consisting of 50 total beds) in our tertiary academic center were asked to fill out a form with their sense of worry for each patient at the start of their shift, and to update it when it changed. These updates were recorded in real time by the nurse taking care of the patient. Shifts are generally 8-hours long, with shifts in the morning, evening, and night. This was recorded using the WF Score form that included the patient’s study identification number, the recording nurse's years of experience, and the date and time. Additionally, nurses collected whether they notified the provider to come to the bedside during a suspected deterioration event, and whether the provider came to the bedside to evaluate the patient. Nurses may have decided to share their WF score when contacting providers, but the WF score was not part of the medical record and frontline providers had not been explicitly informed about the WF score. The nurse’s years of experience was also included to test the hypothesis that nurses with more experience would be more accurate in their judgments of patient risk. The forms were dropped off at the unit desk in a designated location after each shift by the nurses. The charge nurse recorded the number of collected and expected forms. Additionally, charge nurses were asked to independently record their predictions for a subset of patients, to compare with the bedside nurses’ assessment and calculate inter-rater metrics. Charge nurses are experienced nurses that are in charge of overseeing a nursing unit during a specific shift.

### Collection of outcomes and validity data

Data pertaining to resuscitation and RRT calls was obtained from the electronic medical records. Data pertaining to intensive care unit (ICU) transfers was obtained indirectly through the electronic medical records, based on the patient location information. Cases where the WF was 3 or 4, or where the WF was 2 and the nurse requested the provider come to the bedside were considered potential deterioration events and reviewed manually. Each of these cases in which the unit nurse had identified a potential deterioration was evaluated by a team of three reviewers: (1) a physician belonging to the same specialty to which the patient was hospitalized at the time of deterioration but who had no contact with the patient, (2) an experienced nurse who had no contact with the patient, and (3) either a physician, a nurse, or a nurse practitioner familiar with the project who did not have contact with the patient. Reviewers were told to mark an event as a true deterioration if they thought the patient was deteriorating significantly at the time, and if they thought the patient would have benefitted from a bedside assessment by a physician. In each case, the third reviewer assembled a case summary and timeline for Reviewers 1 and 2, but every reviewer also had access to the electronic medical record. Reviews were all performed retrospectively, with every reviewer blinded to the WF score that the nurse had originally assigned. To avoid bias, reviewers were told that control cases had been added. A potential deterioration case was considered a true deterioration if at least two of the three reviewers considered it a true deterioration event. The instances in which the decision was made by a majority vote (two out of the three reviewers as opposed to a unanimous decision), were recorded to track whether a specific reviewer role (e.g., the physician reviewer) was more often the disagreeing vote.

Additionally, outcome events considered included RRT calls, ICU transfers, and codes (call for resuscitation after cardiorespiratory arrest). These outcomes were collected from the medical record.

### Statistical methods

Differences between WF accuracy in nurses with less than 1 year of experience versus more than 1 year of experience was calculated using the chi-squared test. The area under the receiver operating characteristic curve (AUROC) was calculated by treating the WF as an alert: any instance when the WF was above a certain value was treated as an index alert: if the patient had an outcome of interest in the 24 hours after that index alert, it was considered a true positive. If there was no outcome of interest after 24 hours, it was considered a false positive. False negatives were instances in which an outcome occurred that was not preceded by an index alert. True negatives were 24-hour intervals without an outcome or an index alert. Receiver operating characteristic curves (ROC) curves and the area under them (AUROC) for the relationship between WF and ICU transfer in the next 24 hours were calculated two different ways: using the WF as a 5-level variable; and using the WF as an ordinal with three levels: 0 or 1, 2, and 3 or more. Inter-rater reliability was calculated as overall binary agreement between nurse and charge nurse.

Study data were collected and managed using REDCap electronic data capture tools hosted at our institution.^17^ Data presentation and figure construction were done in Microsoft Excel 2010, version 14.0.7177.5000. R version 3.3.1 (Bug in Your Hair) was used for statistical analyses.

## RESULTS

A total of 31 159 patient-shifts were recorded for 3185 unique patients during 3551 hospitalizations. According to the number of expected forms recorded by charge nurses, 93% of expected forms were collected. Patient demographics and length of stay are presented in Table 1. There were 1141 patient-shifts (3.6%) that had one or more increases in the nurse’s WF score. Nurses recorded calling the provider (usually a physician assistant, nurse practitioner, or resident)—either to inform them of the patient’s status or to request them to come assess the patient—a total of 1314 times (1 for every 23 patient-shifts), and recorded the provider coming to the patient’s bedside due to patient deterioration 686 times (1 for every 45 patient-shifts). The number of potential deterioration events identified by nurses was 492 (1% of patient-shifts). Additionally, there were 169 outcome events in total (86 RRT calls, 76 transfers to the ICU, and 7 codes). This data is presented in Table 2.

**Table 1.** Patient demographics and hospitalizations

	All	No events	Events
Patients			
*N*	3185	2972	213
Female. *N* (%)	1600 (50.24%)	1490 (50.13%)	110 (51.64%)
Age. Average (Quartiles)	57.35 (45, 59, 71)	57.21 (44, 59, 71)	59.28 (47, 61, 73)
Hospitalizations			
*N*	3551	3335	216
Length of stay in days. Average (Quartiles)	5.38 (2, 3, 6)	5.05 (2, 3, 6)	10.41 (5, 8, 13.5)

Outcome events included rapid response team calls (RRT calls), ICU transfers, and codes (call for resuscitation after cardiorespiratory arrest).

**Table 2.** Distribution of nursing experience, worry factor scores and outcomes

Nurse’s years of experience	Number of observations
<1 year	2165
1–5 years	16 020
>5 years	12 118
Missing	856
Total	31 159
Worry Factor at the beginning of nursing shift	Number of observations
WF = 0	23 252
WF = 1	6639
WF = 2	613
WF = 3	70
WF = 4	12
Not reported	573
Total number of patient-shifts	31 159
Increased worry factor during the nursing shift	Number of observations
WF = 2	1025
WF = 3	313
WF = 4	92
Total	1430
Distribution of outcomes	Number of observations
Codes	7
ICU transfers	76
RRT calls	86
Deteriorations confirmed by review of those,	380
Deteriorations identified with physician disagreeing	33 (6.7%)
Deteriorations identified with nurse disagreeing	32 (6.5%)
Deteriorations identified with third reviewer disagreeing	52 (10.5%)

### Inter-rater reliability

A total of 611 patient-shifts were evaluated by two nurses (the nurse taking care of the patient and a charge nurse). Overall raw agreement was 0.70, and agreement between 0–1 (not currently deteriorating) and 2–4 (currently deteriorating) was 0.93.

### Worry factor accuracy

**Figure 2.**
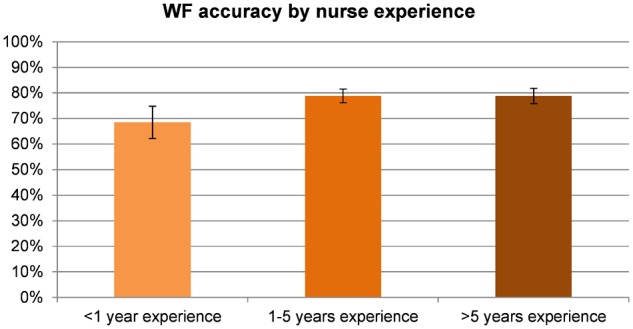
Worry Factor (WF) accuracy by nurse experience. Percentage of potential deteriorations confirmed by reviewers. The graph shows the total percentage of instances that were considered real deterioration by the reviewers, by years of experience of the nurse filling out the worry factor. The difference is statistically significant between less than 1 year of experience, and 1 or more.

Of the 492 potential deterioration events identified by nurses, 380 (77%) were confirmed by reviewers. Reviewer confirmation rates (accuracy of the WF) by nurses’ years of experience are presented in Figure 2. Nurses with less than 1 year of experience had a significantly lower accuracy rate compared to nurses with more than 1 year of experience (68% vs 79%, *P* = 0.04). Baseline risk of deterioration requiring ICU transfer was 0.15% per patient-day; that risk was increased 17-fold (to 2.6% per patient-day) for patients with a WF of 3 or 4, and 38-fold (to 5.8% per patient-day) for patients with a WF of 4. Likelihood ratios for ICU transfer in the following 24 hours were 17.8 (15.2–20.9) for a WF of 3 or 4, and 40.4 (27.1–60.1) for WF of 4. AUROC for transfer to the ICU in the next 24 hours was 0.964 using WF as a 5-level variable, and 0.920 using WF as a three-level ordinal variable.

### Response after increased worry factor during shift

**Figure 3.**
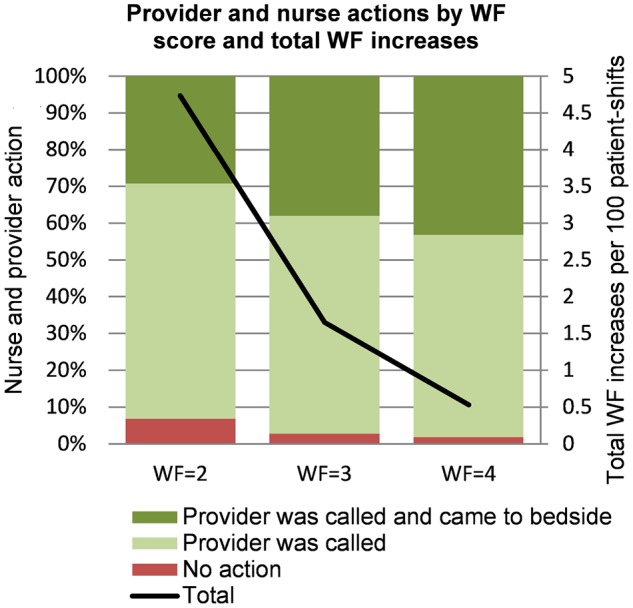
Provider and nurse actions by worry factor (WF) score and number of instances the WF increased during a shift. Number of potential deteriorations and nursing and provider response to increasing worry factor. The graph shows the total number of instances of increased WF by the WF score reached. The line indicates the total number of potential deterioration events (instances of increased worry factor by the nurse). The number of potential deterioration events is smaller for the higher WF scores (more severe deterioration events are rarer). The bars illustrate the proportion of actions taken by the nurse: “no action” means the nurses did not call the provider, “Provider was called” means the provider was notified by the nurse but did not evaluate the patient at the bedside, “Provider was called and came to bedside” means that the provider was notified and performed a bedside evaluation.

Figure 3 shows the actions taken by nurse and provider (after a nurse call) in response to an increase in WF during the shift for WFs of 2, 3, and 4. When the WF was 2, nurses called the provider in 93% of cases, and providers assessed the patient at the bedside in 29% of cases; these proportions increased to 97% and 38%, respectively, for a WF of 3; and to 98% and 43%, respectively, for a WF of 4. The probability of an RRT call taking place in the following 24 hours was 16% after a WF of 2 or above, 37% following a WF of 3 or above, and 63% following a WF of 4.

### Evaluation of the review process

In the deterioration events confirmed by reviewers, we tested the number of times each of the reviewers disagreed to look for role-specific biases (e.g., nurse reviewers finding more potential deterioration events). We found that, of the 380 confirmed deterioration events, the decision was reached with physician reviewer disagreement in 33 cases (6.7%), with nurse disagreement in 32 cases (6.5%), and with third reviewer disagreement in 52 cases (10.5%).

## DISCUSSION

Our study is, to our knowledge, the first to measure the accuracy of nurses’ judgement (which in theory includes both pattern recognition skills and analytical assessment) to detect or predict acute inpatient deterioration with the use of a single score. This was most notably described in Benner’s “Novice to Expert” framework more than 30 years ago.^18^ We found that nurses’ judgement captured through this simple score is predictive of inpatient deterioration: 77% of the potential deterioration events identified by nurses were confirmed by an independent set of reviewers, and a patient with a WF of 3 or more is 40 times more likely to require ICU transfer in the next 24 hours (likelihood ratio = 40.4). This suggests that the nursing staff’s sense of worry, whether through analytical skills or through pattern recognition, is very accurate in identifying deteriorating patients and should be more consistently valued and utilized, considering incorporation into the medical record.

Our current study design doesn’t allow to distinguish whether the accuracy of nursing judgement comes primarily from analytical skills (a conscious and explicit knowledge) or from intuition (an unconscious process relying on pattern recognition). Using Kahneman’s terminology,^19^ we can’t really estimate how much of the WF’s accuracy is from a system 2 (a slow, rational, and effortful analysis based on explicit knowledge), and how much from a system 1 process (a fast, intuitive, and effortless pattern recognition), respectively. The fact that the WF’s AUROC is higher than most published EWS^5^^,^^20–22^ suggests that at least some of its accuracy comes from a pattern recognition process, and not just vital sign information.

In any case, it would not be surprising that nurses develop pattern recognition, since they meet the key characteristics that have been described across multiple fields for the development of pattern recognition^16^: repeated and focused observation of the different patterns to be recognized, as well as rapid and consistent feedback. Nurses generally have more constant and prolonged contact with patients during their hospitalization as compared to physicians, putting them in a particularly advantaged position to recognize patterns that can be a telltale sign of impending physiological deterioration. Nurses frequently use pattern recognition rather than routine vital sign measurement to recognize deterioration in patients, and they often report being able to anticipate a patient’s decline before any objective evidence of deterioration is present.^23^ Based on analytical or pattern recognition processes, our study demonstrates that the WF in its current form is accurate in detecting patient deterioration.

Furthermore, it has recently been shown that educational interventions on nurses may effectively strengthen the afferent limb of a rapid response system,^24^ highlighting the importance of the judgment call by nurses. A retrospective study found that nurse or doctor concern was a significantly more frequent finding in a group of patients before cardiac arrest group than in the control group.^25^ A prospective study found that requiring nurses to assess different changes in a patient’s status, such as changes in their mentation or behavior could contribute to better prediction of unplanned ICU admission or mortality.^26^

Other studies have looked at nurses’ judgement or pattern recognition, referred to in the literature as intuition. Systematic reviews looking at retrospective studies have concluded that intuition plays a significant part in nurses’ detection of deterioration, with vital signs then being used to validate these intuitive feelings,^27^ and that “knowing the patient” is one of four key components in timely recognition of patient deterioration.^27^^,^^28^ The only prospective study on this topic to date used the Dutch-early-nurse-worry-indicator-score (DENWIS),^26^^,^^29^ but it did not include patients from medical wards, and included criteria other than nursing pattern recognition alone. The study concluded that DENWIS improved calling criteria based on vital signs.^26^ Of the aforementioned studies, ours is the only study that quantifies deteriorations that are resolved on the ward (including medical wards) without an ICU transfer, and focuses on general nursing judgement including analytical skills as well as subjective worry or pattern recognition.

The WF score can provide a common language to clearly and succinctly communicate priority and perceived urgency. It is, in that regard, similar to frameworks like the I-PASS handoff bundle,^30^ which classifies patients as either stable, unstable, or watcher. The WF score provides one additional level of detail by using three levels for the “unstable” category (WF scores of 2, 3, or 4), and we have demonstrated its accuracy when used by nursing staff. The scale used is numerical and easy to pull into EWS calculations. Additionally, we encountered anecdotal evidence that implementation of the WF can increase nurse satisfaction by improving communication.

We also looked into whether nursing years of experience improved the accuracy of the WF. Nurses with less than 1 year of experience seem to be less accurate than nurses with more than 1 year of experience (68% vs 79%, *P* = 0.04) Additionally, we anticipated that nurses reviewing potential deterioration cases would tend to sympathize with the worried nurse requesting help from a provider and, consequently be more likely to classify cases as true deteriorations. Conversely, we expected physician reviewers to tend to classify cases less often as true deteriorations requiring bedside assessment, having personally experienced instances when they were called with urgency to the bedside to find that they were not truly required. We found no evidence of this role-dependent bias, with no significant difference in the number of cases in which physicians and nurses disagreed (6.7% and 6.5%, respectively).

This study is not without limitations. Our retrospective review may have missed some deterioration events that were missed by the nurse and did not eventually result in an RRT call, an ICU transfer or a code. On the other hand, our accuracy numbers are likely a conservative estimate given that reviewers were told that some control cases (non-deterioration cases) were included in the review. Data collection was conducted in a single tertiary referral center that hires carefully selected and highly trained registered nurses. The accuracy of these WF scores could change if conducted in a different center, where nurses might have different skillsets. Before the WF can be implemented in the hospital, some key design questions would need to be answered, for example where in the medical record it would be displayed. Another question is whether to include a specific policy expecting a bedside assessment at a certain WF level, or to leave it to the care team’s discretion as was done in our study. Using the WF as an element in an EWS would also require additional analysis to determine the weight of the WF in relationship with other elements of the EWS. It would also be of interest to study whether a nurse education program is able to improve accuracy of the WF score.

Our results highlight the importance of the information acquired directly at the patient’s bedside and of the nursing assessment, so we would suggest an implementation strategy that used the WF in combination with an EWS, and included in some capacity a requirement for the care team to meet at the patient’s bedside for a team evaluation and discussion of the patient’s condition.

## CONCLUSIONS

The results of our study show that a simple WF score, based on nurses’ pattern recognition is able to accurately predict patient deterioration in a hospital setting. This simple score could be used alone or easily incorporated into existing EWS to potentially improve their performance. However, our results also raise the need for further research, including design questions before the score can be implemented in practice to determine whether patient outcomes can be improved through its application.

## DECLARATIONS

### Consent for publication

No individual patient data is included in the manuscript.

### Availability of data and material

The data is available upon request after review and approval from the author's relevant institutional committees.

## FUNDING

The study was funded using internal Mayo Clinic funds. This publication was made possible by CTSA Grant Number UL1 TR000135 from the National Center for Advancing Translational Sciences (NCATS), a component of the National Institutes of Health (NIH). Its contents are solely the responsibility of the authors and do not necessarily represent the official view of NIH.

## AUTHORS’ CONTRIBUTIONS

SRB and KG conceptualized the study; SRB, KG and JH designed the methodology and performed the investigation; SRB, JH and MGJ performed the formal data analysis; SRB and CN prepared the original draft; KG, JH, MGJ and JMH reviewed and edited the manuscript. All authors reviewed and approved the final version to be submitted.

## ETHICS APPROVAL AND CONSENT TO PARTICIPATE

Data collection was approved by Mayo Clinic’s Institutional Review Board (IRB number 13-004945). Consent was waived by the IRB.

## References

[1] Liu V, Escobar GJ, Greene JD, et al Hospital deaths in patients with sepsis from 2 independent cohorts. JAMA 2014; 312 (1): 90–2. 24838355 10.1001/jama.2014.5804

[2] Kumar A, Roberts D, Wood KE, et al Duration of hypotension before initiation of effective antimicrobial therapy is the critical determinant of survival in human septic shock. Crit Care Med 2006; 34 (6): 1589–96. 16625125 10.1097/01.CCM.0000217961.75225.E9

[3] Young MP, Gooder VJ, McBride K, et al Inpatient transfers to the intensive care unit: delays are associated with increased mortality and morbidity. J Gen Intern Med 2003; 18 (2): 77–83. 12542581 10.1046/j.1525-1497.2003.20441.x PMC1494814

[4] Chalfin DB, Trzeciak S, Likourezos A, et al Impact of delayed transfer of critically ill patients from the emergency department to the intensive care unit. Crit Care Med 2007; 35 (6): 1477–83. 17440421 10.1097/01.CCM.0000266585.74905.5A

[5] Romero-Brufau S, Huddleston JM, Naessens JM, et al Widely used track and trigger scores: are they ready for automation in practice? Resuscitation 2014; 85 (4): 549–52. 24412159 10.1016/j.resuscitation.2013.12.017

[6] Alam N, Hobbelink EL, van Tienhoven AJ, et al The impact of the use of the Early Warning Score (EWS) on patient outcomes: a systematic review. Resuscitation 2014; 85 (5): 587–94. 24467882 10.1016/j.resuscitation.2014.01.013

[7] Alam N, Vegting IL, Houben E, et al Exploring the performance of the National Early Warning Score (NEWS) in a European emergency department. Resuscitation 2015; 90: 111–5. 25748878 10.1016/j.resuscitation.2015.02.011

[8] Nishijima I, Oyadomari S, Maedomari S, et al Use of a modified early warning score system to reduce the rate of in-hospital cardiac arrest. J Intensive Care 2016; 4 (1): 12. 26865981 10.1186/s40560-016-0134-7 PMC4748572

[9] Chan PS, Jain R, Nallmothu BK, et al Rapid response teams: a systematic review and meta-analysis. Arch Intern Med 2010; 170 (1): 18–26. 20065195 10.1001/archinternmed.2009.424

[10] Jones DA, DeVita MA, Bellomo R. Rapid-response teams. N Engl J Med 2011; 365 (2): 139–46. 21751906 10.1056/NEJMra0910926

[11] Winters BD, Weaver SJ, Pfoh ER, et al Rapid-response systems as a patient safety strategy. Ann Intern Med 2013; 158(5 Pt 2): 417–25. 23460099 10.7326/0003-4819-158-5-201303051-00009 PMC4695999

[12] Finlay GD, Rothman MJ, Smith RA. Measuring the modified early warning score and the Rothman index: advantages of utilizing the electronic medical record in an early warning system. J Hosp Med 2014; 9 (2): 116–9. 24357519 10.1002/jhm.2132 PMC4321057

[13] Rothman MJ, Rothman SI, Beals J. Development and validation of a continuous measure of patient condition using the Electronic Medical Record. J Biomed Inform 2013; 46 (5): 837–48. 23831554 10.1016/j.jbi.2013.06.011

[14] Genardi ME, Cronin SN, Thomas L. Revitalizing an established rapid response team. Dimens Crit Care Nurs 2008; 27 (3): 104–9. 18434864 10.1097/01.DCC.0000286837.95720.8c

[15] Santiano N, Young L, Hillman K, et al Analysis of medical emergency team calls comparing subjective to “objective” call criteria. Resuscitation 2009; 80 (1): 44–9. 18952358 10.1016/j.resuscitation.2008.08.010

[16] Kahneman D, Klein G. Conditions for intuitive expertise: a failure to disagree. Am Psychol 2009; 64 (6): 515–26. 19739881 10.1037/a0016755

[17] Harris PA, Taylor R, Thielke R, et al Research electronic data capture (REDCap)–a metadata-driven methodology and workflow process for providing translational research informatics support. J Biomed Inform 2009; 42 (2): 377–81. 18929686 10.1016/j.jbi.2008.08.010 PMC2700030

[18] Benner P. From novice to expert. Am J Nurs 1982; 82 (3): 402–7. 6917683

[19] Kahneman D. *Thinking, Fast and Slow* 1st pbk ed. New York: Farrar, Straus and Giroux; 2013.

[20] Smith GB, Prytherch DR, Meredith P, et al The ability of the National Early Warning Score (NEWS) to discriminate patients at risk of early cardiac arrest, unanticipated intensive care unit admission, and death. Resuscitation 2013; 84 (4): 465–70. 23295778 10.1016/j.resuscitation.2012.12.016

[21] Smith ME, Chiovaro JC, O’Neil M, et al Early warning system scores for clinical deterioration in hospitalized patients: a systematic review. Annals Am Thorac Soc 2014; 11 (9): 1454–65. 10.1513/AnnalsATS.201403-102OC 25296111

[22] Smith GB, Prytherch DR, Schmidt PE, et al Review and performance evaluation of aggregate weighted ‘track and trigger’ systems. Resuscitation 2008; 77 (2): 170–9. 18249483 10.1016/j.resuscitation.2007.12.004

[23] Douw G, Schoonhoven L, Holwerda T, et al Nurses' worry or concern and early recognition of deteriorating patients on general wards in acute care hospitals: a systematic review. Crit Care 2015; 19: 230. 25990249 10.1186/s13054-015-0950-5 PMC4461986

[24] Liaw SY, Wong LF, Ang SB, et al Strengthening the afferent limb of rapid response systems: an educational intervention using web-based learning for early recognition and responding to deteriorating patients. BMJ Qual Saf 2016; 25 (6): 448–56. 10.1136/bmjqs-2015-004073 26297379

[25] Hodgetts TJ, Kenward G, Vlachonikolis IG, et al The identification of risk factors for cardiac arrest and formulation of activation criteria to alert a medical emergency team. Resuscitation 2002; 54 (2): 125–31. 12161291 10.1016/s0300-9572(02)00100-4

[26] Douw G, Huisman-de Waal G, van Zanten AR, et al Nurses' ‘worry’ as predictor of deteriorating surgical ward patients: a prospective cohort study of the Dutch-Early-Nurse-Worry-Indicator-Score. Int J Nurs Stud 2016; 59: 134–40. 27222458 10.1016/j.ijnurstu.2016.04.006

[27] Odell M, Victor C, Oliver D. Nurses' role in detecting deterioration in ward patients: systematic literature review. J Adv Nurs 2009; 65 (10): 1992–2006. 20568317 10.1111/j.1365-2648.2009.05109.x

[28] Massey D, Chaboyer W, Anderson V. What factors influence ward nurses' recognition of and response to patient deterioration? An integrative review of the literature. Nurs Open 2017; 4 (1): 6–23. 28078095 10.1002/nop2.53 PMC5221430

[29] Douw G, Huisman-de Waal G, van Zanten AR, et al Capturing early signs of deterioration: the dutch-early-nurse-worry-indicator-score and its value in the Rapid Response System. J Clin Nurs 2016; 10.1111/jocn.13648 27865003

[30] Starmer AJ, O’Toole JK, Rosenbluth G, et al Development, implementation, and dissemination of the I-PASS handoff curriculum: A multisite educational intervention to improve patient handoffs. Acad Med 2014; 89 (6): 876–84. 24871238 10.1097/ACM.0000000000000264

